# Traumatic Dental Injuries at a Tertiary Dental Trauma Center in Eastern Bavaria: Findings from the Regensburg Dental Trauma Registry

**DOI:** 10.3390/jcm15207947

**Published:** 2026-10-14

**Authors:** Sevgi Arabulan, Karolina Müller, Gunnar Huppertz, Martyna Smeda, Michael Koller, Sophia Rebecca Lindner, Torsten E. Reichert, Miriam Hergert, Johanna Maluche, Wolfgang Buchalla, Matthias Widbiller

**Affiliations:** 1 Department of Conservative Dentistry and Periodontology, University Hospital Regensburg, 93053 Regensburg, Germany; sevgi.arabulan@klinik.uni-regensburg.de (S.A.);; 2 Center for Clinical Studies, University Hospital Regensburg, 93053 Regensburg, Germany; 3 Department of Cranio-Maxillofacial Surgery, University Hospital Regensburg, 93053 Regensburg, Germany

**Keywords:** dental trauma, tooth injuries, traumatology, registries, tooth avulsion, tooth luxation, tooth fractures

## Abstract

**Background:** Traumatic dental injuries (TDIs) are highly prevalent worldwide; however, structured and standardized datasets remain scarce. This study aimed to describe the demographics, injury characteristics, diagnostic findings, and initial management of TDIs in Eastern Bavaria using data from the Regensburg Dental Trauma Registry (RDTR). **Methods**: This single-center, retrospective study included 1000 cases with TDIs presented within three days after trauma and treated at the Center for Dental Traumatology, University Hospital of Regensburg, between June 2016 and December 2020, representing the initial cohort of the ongoing RDTR. Data were recorded in a standardized way using a REDCap-based electronic case report form. Descriptive analyses were performed at both the case and tooth levels. **Results:** The cohort comprised 1000 cases (61.6% male, 38.4% female; median age 13 years [IQR: 7–26]), with a total of 1904 permanent and 528 primary teeth affected. A total of 84.1% presented within 24 h of injury. The age distribution of presenting cases peaked at 2–4 and 7–9 years, irrespective of sex. The relative distributions were similar among children aged ≤3 years. Age groups between 4 and 27 years accounted for larger proportions of male than female registry cases, whereas age groups ≥28 years accounted for larger proportions of female than male registry cases. Falls were the leading cause of injury (67.8%), with cycling being the most common activity at the time of trauma (18.6%). Maxillary central incisors were the most frequently affected teeth in both dentitions (58.7% primary; 54.9% permanent). Luxation injuries predominated in primary teeth (92.8%) compared with permanent teeth (69.8%), whereas fractures were more common in permanent teeth (57% vs. 14.2%). Avulsion injuries affected 202 teeth (139 permanent, 63 primary). Among cases involving at least one avulsed permanent tooth, dry storage was documented in 40.9% of cases. The most frequent initial treatments were splinting (36.5%), restoration or coverage of fractured surfaces (27.0%), and repositioning (11.3%). **Conclusions**: Standardized registry-based documentation can provide a comprehensive basis for understanding the clinical presentation and management of traumatic dental injuries and for identifying opportunities to improve trauma care. The RDTR establishes a framework for systematic data collection that can support quality improvement, comparative research, and longitudinal evaluation of treatment outcomes. Its continued development and expansion across centers may contribute to a more robust evidence base for the management of TDIs.

## 1. Introduction

Traumatic dental injury (TDI) is common and may result in functional and esthetic impairments, which can lead to social and economic consequences and may adversely affect quality of life in the long term [1].

TDIs can occur at any age but are more prevalent in children and adolescents, with males affected more frequently [2,3,4]. Both primary and permanent teeth may be involved, and it is estimated that nearly one billion people worldwide have experienced at least one TDI during their lifetime [5]. Although TDIs represent a global health issue, epidemiological studies report substantial variability in prevalence rates, ranging from 4.1% to 58.6%, reflecting differences in population characteristics, reporting practices, and methodological approaches [6].

Beyond their impact on individuals, TDIs impose a substantial burden on healthcare systems and society, underscoring their relevance as a public health concern [7]. Management often requires complex long-term treatment, resulting in considerable healthcare costs and productivity losses [8]. Furthermore, dental trauma places a sustained strain on public health insurance systems due to the need for long-term maintenance and management of late complications [9]. Therefore, identifying risk factors and implementing effective preventive strategies is essential for reducing both the incidence of TDIs and their long-term economic impact.

Despite the clear epidemiological relevance of TDIs, robust and structured data collection remains limited, and dedicated registries are scarce [10]. This lack of standardization is also evident in Germany, where the literature is largely based on modest cohort studies [11,12], while large-scale analyses remain scarce [13,14]. Furthermore, heterogeneity in reporting standards limits comparability and contributes to a fragmented understanding of TDI epidemiology. Standardized, large-scale datasets are therefore essential for analyzing trauma-related parameters and optimizing evidence-based clinical management of TDIs.

Recognizing the need for standardized TDI documentation and specialized emergency trauma care, the University Hospital Regensburg established the Center for Dental Traumatology in 2016. The center provides 24/7 emergency care for acute dental trauma across eastern Bavaria. In parallel, the Regensburg Dental Trauma Registry (RDTR) was established to facilitate systematic and standardized data collection, as previously described [15]. Using data from the registry, this study aimed to describe the demographics, injury characteristics, diagnostic findings, and initial management of TDIs in Eastern Bavaria.

## 2. Materials and Methods

### 2.1. Study Design and Data Collection

This single-center registry study was conducted within the context of the RDTR and approved by the Ethics Committee of the University of Regensburg (No. 24-3939-104). The study was reported in accordance with the REporting of studies Conducted using Observational Routinely collected health Data (RECORD) statement [16] (Supplementary Table S1). All cases presenting to the Center for Dental Traumatology at the University Hospital Regensburg with an acute TDI were systematically registered in the RDTR. The investigators had access to the complete RDTR dataset for the predefined study period.

The study included the first 1000 eligible cases registered in the RDTR who presented to the Center for Dental Traumatology at the University Hospital Regensburg, which was predefined in the RDTR project plan as the initial study cohort. Each traumatic event was recorded and analyzed as a separate case. TDI documentation was conducted according to the methodological framework of the RDTR as previously described [15]. A standardized documentation form, based on national treatment guidelines [17], was implemented to systematically record demographic and trauma-related information derived from anamnesis, clinical examination and radiographic findings. TDIs were classified according to the Andreasen classification [18]. To account for trauma-related damage to pre-existing restorations, “damage to fillings” and “damage to prosthetic restorations” were also recorded.

### 2.2. Data Management and Quality Assurance

Documentation forms were independently reviewed and transferred into an electronic database (REDCap) by two trained dentists according to a standardized data transfer manual to guarantee reproducibility.

Data cleaning was performed to ensure data quality. Therefore, predefined plausibility checks on internal consistency, logical dependencies between variables, and clinical plausibility were applied. Descriptive interim analyses were conducted to identify any outliers and missing values. When inconsistencies or implausible entries were detected, the original patient records and available radiographs were reviewed and corrected. Inconsistencies that could not be resolved were excluded from further analyses. A detailed overview of the plausibility checks and the corresponding exclusions is provided in Supplementary Table S2. Cases with ongoing registration and cases with no traumatized teeth were excluded.

**Figure 1:**
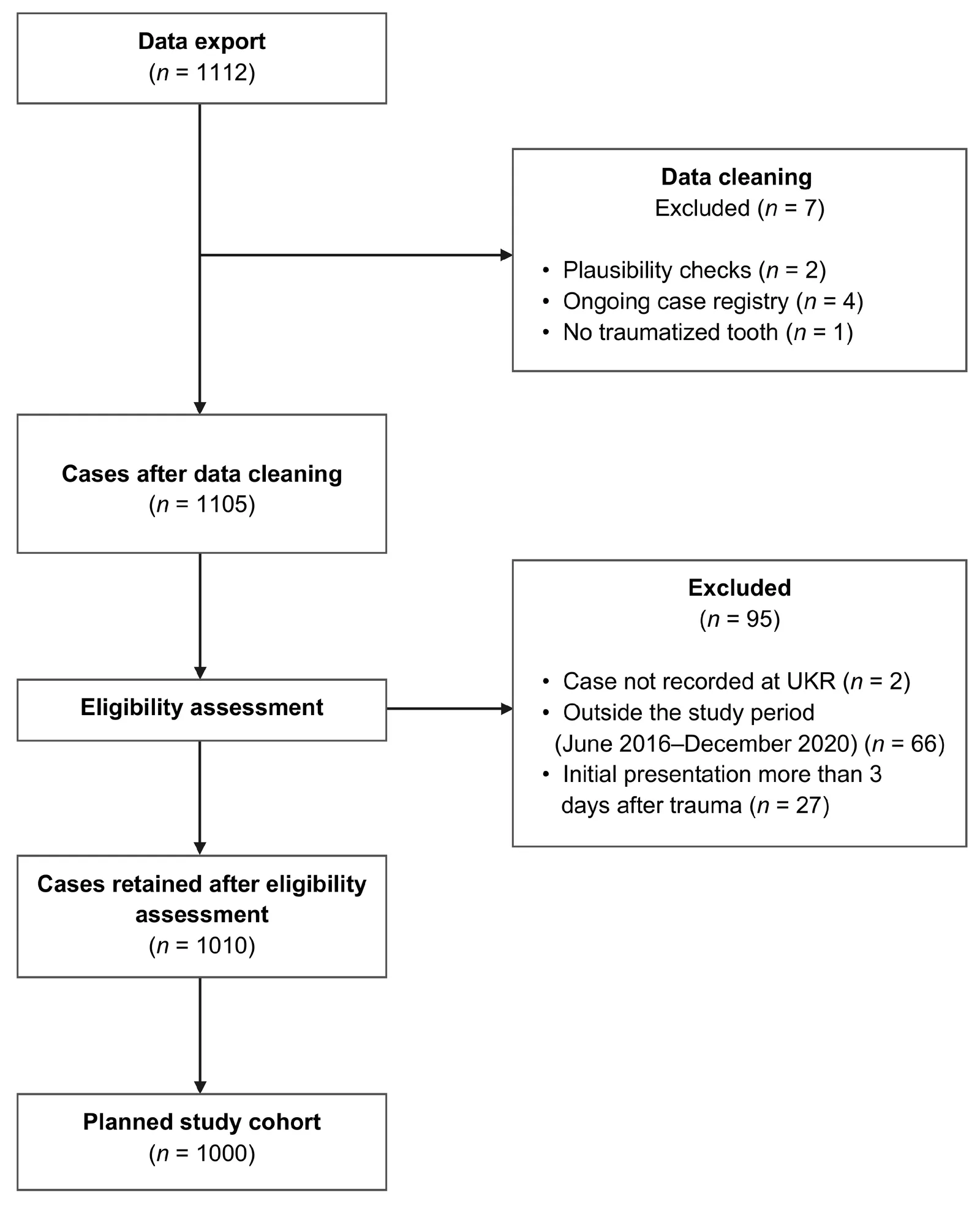
Flow diagram illustrating the selection of the initial study cohort.

After data cleaning, the inclusion and exclusion criteria were applied. The inclusion criteria were as follows: (1) patients presenting to University Hospital Regensburg; (2) cases recorded between June 2016 and December 2020; and (3) cases presenting within three days of the traumatic event. The case selection process is summarized in Figure 1.

Following data cleaning and eligibility assessment, 1010 cases remained eligible. As predefined in the RDTR project plan, the first 1000 cases listed in chronological order were included in the analyses. The remaining 10 eligible cases, all of which were registered in December 2020, were not included. The 1000 cases were contributed by 979 unique patients, with 21 cases representing recurrent TDIs.

### 2.3. Data Analysis

Data were analyzed at both the case and tooth levels, according to the nature of the variable. The case level was defined by the traumatic event and was used for, e.g., patient demographics, trauma circumstances, temporal characteristics, and associated medical findings. Recurrent TDIs affecting the same patient were recorded and analyzed as separate cases; thus, patients with recurrent injuries contributed one case for each traumatic event. Tooth-level analyses were used for variables describing individual affected teeth, including tooth type, injury diagnosis, and treatment measures.

Demographic variables included patient sex and age. Age was categorized in six groups: ≤3 years (infants or toddlers), 4–12 years (children), 13–18 years (adolescents), 19–27 years (young adults), 28–65 years (adults), ≥66 years (older adults). Age groups were defined a priori as mutually exclusive and exhaustive categories, based primarily on established legal and health-related age classifications in Germany. Additional boundaries were introduced where appropriate to reflect relevant developmental and epidemiological transitions. This approach ensured complete, non-overlapping categories while providing a clinically meaningful representation of different life stages.

Temporal variables of trauma included season, day of the week, time of day, and the interval between the traumatic event and initial clinic presentation. Trauma circumstances were characterized by the activity at the time of injury, mechanism of injury, and location of occurrence.

Trauma-related medical findings recorded at presentation included neurological indicators suggestive of potential traumatic brain injury, such as loss of consciousness, nausea or vomiting, and retrograde amnesia.

Injury-related variables included affected teeth, diagnostic findings, and the presence of soft tissue or alveolar process involvement. Injuries were further stratified by primary and permanent teeth. Trauma-specific parameters included extent and direction of displacement (mm) in luxation injuries and fragment status in fracture cases. For avulsion injuries, storage condition and extraoral time were recorded at the case level. When multiple teeth were avulsed within the same case, the documented storage condition and extraoral time were therefore applied to all avulsed teeth within that case. Analyses of storage condition and extraoral time were restricted to cases involving avulsion of at least one permanent tooth.

Initial treatment measures, including restorative procedures, repositioning, replantation and splinting, were recorded at the tooth level.

### 2.4. Data Presentation

Data were analyzed descriptively at both case and tooth levels. Categorical variables are presented as absolute numbers (n) and percentages (%), while continuous variables are reported as mean ± standard deviation (SD) for approximately normally distributed data or median with interquartile range (IQR) for non-normally distributed data. All analyses were performed using SPSS software package (version 29.0, IBM Corp., Armonk, NY, USA).

## 3. Results

### 3.1. Demographic Variables

**Figure 2:**
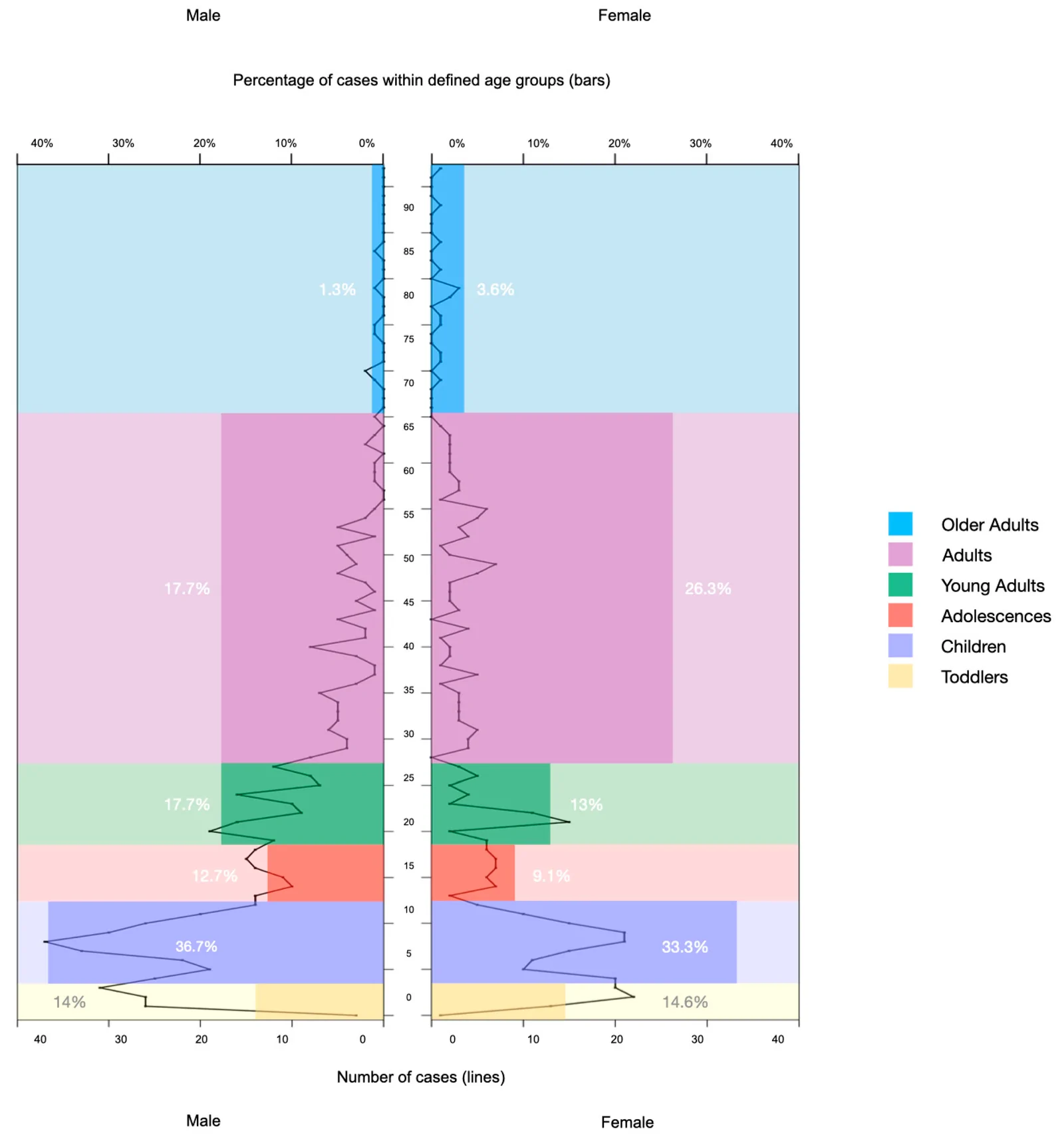
Distribution of TDIs by age and sex. Age-group percentages are represented by horizontal bars (males on the left, females on the right; upper x-axis) across predefined age groups. Superimposed line graphs indicate the absolute number of cases by single year of age (lower x-axis) against age in years (y-axis).

The study population comprised 61.6% male and 38.4% female cases. The median age was 12 years (IQR: 7–24) in males and 14 years (IQR: 6–34) in females (Figure 2). Children aged 4–12 years constituted the largest age group, accounting for 36.7% of males and 33.3% of females (Figure 2). Sex differences were minimal among toddlers, whereas males predominated in the child, adolescent, and young adult age groups, with differences of 3.4%, 3.6%, and 4.7%, respectively. In contrast, the adult and older adult groups accounted for a larger proportion of female registry cases than male registry cases, with differences of 8.6 and 2.3 percentage points, respectively.

### 3.2. Temporal Variables

The largest proportion of injuries occurred during the meteorological summer months (June–August; *n* = 210, 28.3%), followed by spring (March–May; *n* = 197, 26.6%) and autumn (September–November; *n* = 184, 24.8%). Fewer cases occurred during winter (December–February; *n* = 150, 20.2%). Monthly and seasonal analyses were restricted to cases that occurred during the three fully documented calendar years, 2017–2019 (*n* = 741). The year 2016 was excluded because registry documentation began in June, and 2020 was excluded because 10 eligible cases from December occurred beyond the predefined case limit. The year-specific seasonal distribution for the included calendar years (2017–2019) is presented in Supplementary Table S3.

Most traumatic injuries were registered on Fridays and during weekends (66.3%), compared with 33.7% between Monday and Thursday (Supplementary Figure S1A). The highest frequency of TDIs was observed in the late afternoon and early evening, between 3:00 p.m. and 8:00 p.m., accounting for 41.4% of cases (Supplementary Figure S1B). No information on the time of injury was available for 108 cases. Details on missing information for key variables at the case and tooth levels are provided in Supplementary Table S4.

Overall, 84.1% of cases presented within 24 h of sustaining a TDI, whereas 2.9% presented between 24 and 72 h; for 13% of cases, no information was available. Among cases with documented presentation times, 80.2% presented within the first five hours, with most patients seeking care between one and two hours (26.4%) after the traumatic incident (Supplementary Figure S2).

### 3.3. Trauma Circumstances

**Figure 3:**
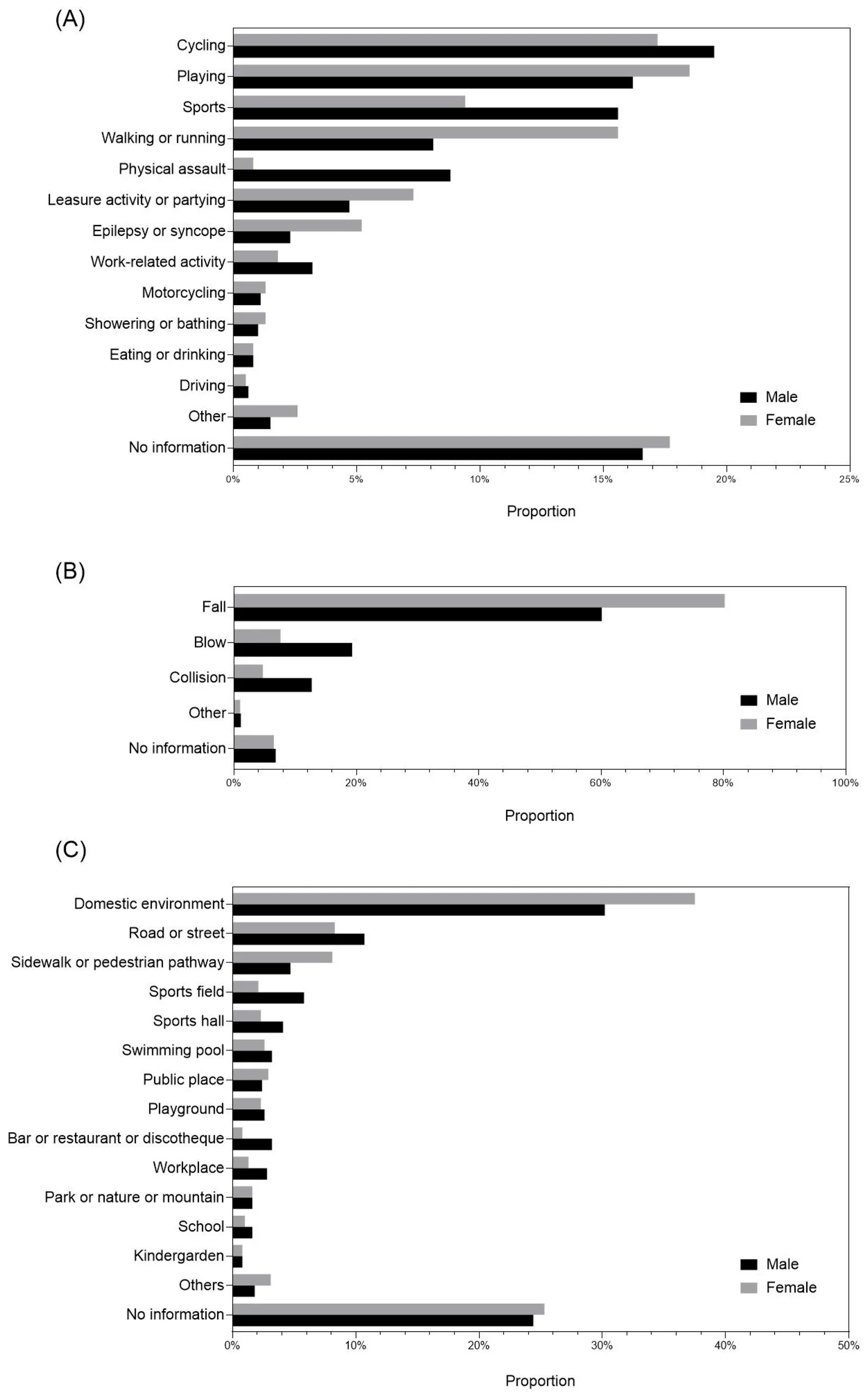
Distribution of traumatic dental injuries according to (**A**) activity at the time of trauma, (**B**) cause of trauma, and (**C**) location of injury. Percentages are broken down by sex for each clinical parameter.

Among activities performed at the time of injury, bicycle riding accounted for the highest proportion of cases (18.6%), followed by playing activities (17.1%) and walking or running (11.0%) (Figure 3A). For selected categories comprising at least 20 cases (≥2% of the cohort), unadjusted descriptive proportion ratios were calculated by dividing the proportion of male registry cases in a given category by the corresponding proportion of female registry cases. The corresponding case counts, sex-specific proportions, and descriptive proportion ratios are presented in Supplementary Table S5. These ratios were used only to describe the relative distribution of characteristics among male and female registry cases and should not be interpreted as population-level risk or frequency ratios.

Sports-related injuries, work-related accidents, and physical assaults accounted for larger proportions of male than female registry cases, with unadjusted descriptive proportion ratios of 1.7, 1.8, and 11.0, respectively. Conversely, walking- or running-related injuries and syncope-related events accounted for larger proportions of female than male registry cases, with corresponding ratios of 1.9 and 2.3, respectively.

Falls were the predominant mechanism of injury (67.8%), followed by blows (14.8%) and collisions (9.6%) (Figure 3B). Falls accounted for a larger proportion of female than male registry cases, with an unadjusted descriptive proportion ratio of 1.3. In contrast, injuries caused by blows and collisions accounted for larger proportions of male registry cases, with corresponding ratios of 2.5 and 2.7, respectively.

Most injuries occurred in the domestic environment (33.0%), followed by roads or streets (9.8%) and sidewalks or pedestrian pathways (6.0%) (Figure 3C). Injuries occurring in the domestic environment and on sidewalks or pedestrian pathways accounted for larger proportions of female than male registry cases, with unadjusted descriptive proportion ratios of 1.2 and 1.7, respectively. Conversely, injuries occurring at workplaces, on sports fields, and in bars or restaurants accounted for larger proportions of male than female registry cases, with corresponding ratios of 2.2, 2.8, and 4.0, respectively.

### 3.4. Trauma-Related Medical Findings

Overall, trauma-related medical findings at presentation were not uncommon. Symptoms suggestive of traumatic brain injury included loss of consciousness in 6.9% of cases, nausea or vomiting in 5.7%, and retrograde amnesia in 4.9%. Patients with reported symptoms or signs potentially suggestive of head injury were referred to the hospital’s emergency department for further medical assessment; data from these subsequent medical evaluations were not included in the present study.

### 3.5. Distribution of Affected Teeth

Of the 2432 traumatized teeth from the first 1000 cases, 1904 were permanent and 528 were primary. Maxillary central incisors were the most frequently affected teeth in both dentitions (55.8%), followed by maxillary lateral incisors (22.7%) and mandibular incisors (6.6%; Supplementary Figure S3).

With permanent teeth, the affected teeth were almost equally distributed between the right (49.8%) and left (50.2%) quadrants. A similar distribution was observed in primary teeth, with 50.4% of the affected teeth located in the right quadrants, while 49.6% in the left quadrants, indicating no relevant side predominance.

### 3.6. Diagnostic Findings

Following injury, tooth-level analyses showed that pulp sensibility testing to cold yielded negative responses in 11.2% of traumatized permanent teeth and 6.8% of primary teeth; corresponding information was unavailable for 24.7% and 69.9%, respectively. Among teeth with available test results, pulp sensibility testing to cold was positive in 85.1% of permanent teeth and 77.4% of primary teeth, and negative in 14.9% and 22.6%, respectively. Percussion testing yielded positive responses in 42.9% of permanent teeth and 26.5% of primary teeth, while negative responses were recorded in 33.5% and 19.7%, respectively. Missing information was more frequent for primary teeth (53.8%) than for permanent teeth (23.6%). Among teeth with available test results, percussion testing was positive in 56.1% of permanent teeth and 57.4% of primary teeth, and negative in 43.9% and 42.6%, respectively. Missing information primarily resulted from insufficient patient cooperation, particularly among young children, diagnostic assessments in which no clearly interpretable result could be obtained, or incomplete documentation (Supplementary Table S6).

Case-based analysis showed that 45.9% of cases did not undergo radiographic imaging at the time of initial presentation. Orthopantomograms (OPGs) were obtained in 28.8% of cases, while periapical radiographs were performed in 29.4%. Cone-beam computed tomography (CBCT) was rarely used in the acute setting (0.1%). Additional in-house imaging techniques were used in 1.8% of cases, and external radiographs were available for 1.0% of cases. In 6.9% of the cases, more than one imaging modality was used; therefore, the reported categories were not mutually exclusive. An age-stratified distribution of imaging modalities is presented in Supplementary Table S7 and demonstrates a more selective use of radiographic imaging in younger children, with imaging being used less frequently as age decreased.

### 3.7. Soft Tissue and Alveolar Process Injuries

Additional soft tissue injuries were documented in 63.1% of cases. Isolated extraoral injuries were observed in 26.1% of cases, whereas isolated intraoral injuries occurred in 9.8%. Combined extraoral and intraoral soft tissue injuries were present in 27.2% of cases. No soft tissue injury was documented in 36.9% of the cases.

Alveolar process fractures were identified in 6.7%, while fracture status was undocumented in 0.9%. Among these fractures, 77.6% involved the maxilla and 22.4% the mandible.

### 3.8. Trauma Type

Luxation injuries predominated in both primary and permanent teeth, particularly in primary teeth. Fractures were observed in 57.0% of permanent teeth and 14.2% of primary teeth (Supplementary Figure S4), whereas luxation injuries accounted for 69.8% of traumatized permanent teeth and 92.8% of traumatized primary teeth. Because a single tooth could sustain both fracture and luxation injuries, these diagnostic categories were not mutually exclusive; therefore, their percentages do not sum to 100%.

Overall, approximately half of all traumatized teeth (47.7%) exhibited a fracture. Among fractured teeth, crown fractures were the most common injury type (64.5%). In both dentitions, crown fractures without pulp involvement predominated, accounting for 53.6% of fractures in permanent teeth and 34.7% in primary teeth. Although uncomplicated crown fractures predominated in both dentitions, pulp involvement was relatively more frequent compared with uncomplicated crown fractures in primary teeth (13.3% vs. 34.7%) than in permanent teeth (12.0% vs. 53.6%). Root fractures were also observed more frequently in primary teeth (Table 1).

Among all luxation injuries, subluxation was the most common type in both dentitions, accounting for 40.3% of cases overall. In primary teeth, subluxation was followed by lateral luxation (26.9%) and avulsion (12.9%). In contrast, in permanent teeth, subluxation was followed by concussion (26.7%) and lateral luxation (14.2%). Concussion injuries were substantially more frequent in permanent teeth, whereas lateral luxations were more common in primary teeth. Information required to classify the specific type of luxation was missing in only a small proportion of cases in both dentitions (2.5% in permanent teeth and 4.3% in primary teeth) (Table 1).

### 3.9. Luxation-Specific Parameters

Among teeth diagnosed with subluxation, Grade I mobility was most common (68.7% in permanent teeth and 42.8% in primary teeth). Higher mobility (Grades II to III) was observed more frequently in primary than in permanent teeth with subluxation (Supplementary Figure S5).

The extent of luxation displacement varied by injury type and dentition. In permanent teeth, median displacement was 2 mm (IQR: 1–3 mm) for lateral luxation (*n* = 162), 2 mm (IQR: 1–3.25 mm) for extrusion (*n* = 34), and 3 mm (IQR: 1.5–10 mm) for intrusion (*n* = 17). Corresponding values in primary teeth were 1 mm (IQR: 1–2 mm) for lateral luxation (*n* = 102), 3 mm (IQR: 1.25–4 mm) for extrusion (*n* = 16), and 2.5 mm (IQR: 1.25–3 mm) for intrusion (*n* = 20) (Supplementary Figure S6).

Most luxated teeth were displaced orally (73.0% of permanent teeth and 71.2% of primary teeth), followed by vestibular displacement (14.8% and 15.9%, respectively). Mesial (0.5% and 1.5%, respectively) and distal (0.5% and 0%, respectively) displacements were rare.

### 3.10. Avulsion-Specific Parameters

Of the 1000 cases, 144 involved tooth avulsions, affecting 202 teeth in total (63 primary and 139 permanent). Of these 144 cases, 92 involved permanent teeth only, 51 involved primary teeth only, and one involved both permanent and primary teeth. The subsequent analysis of storage conditions was restricted to the 93 cases involving avulsion of at least one permanent tooth. In 76 cases (81.7%), at least one avulsed permanent tooth was brought to the clinic, whereas in 17 cases (18.3%), no avulsed tooth was presented.

**Figure 4:**
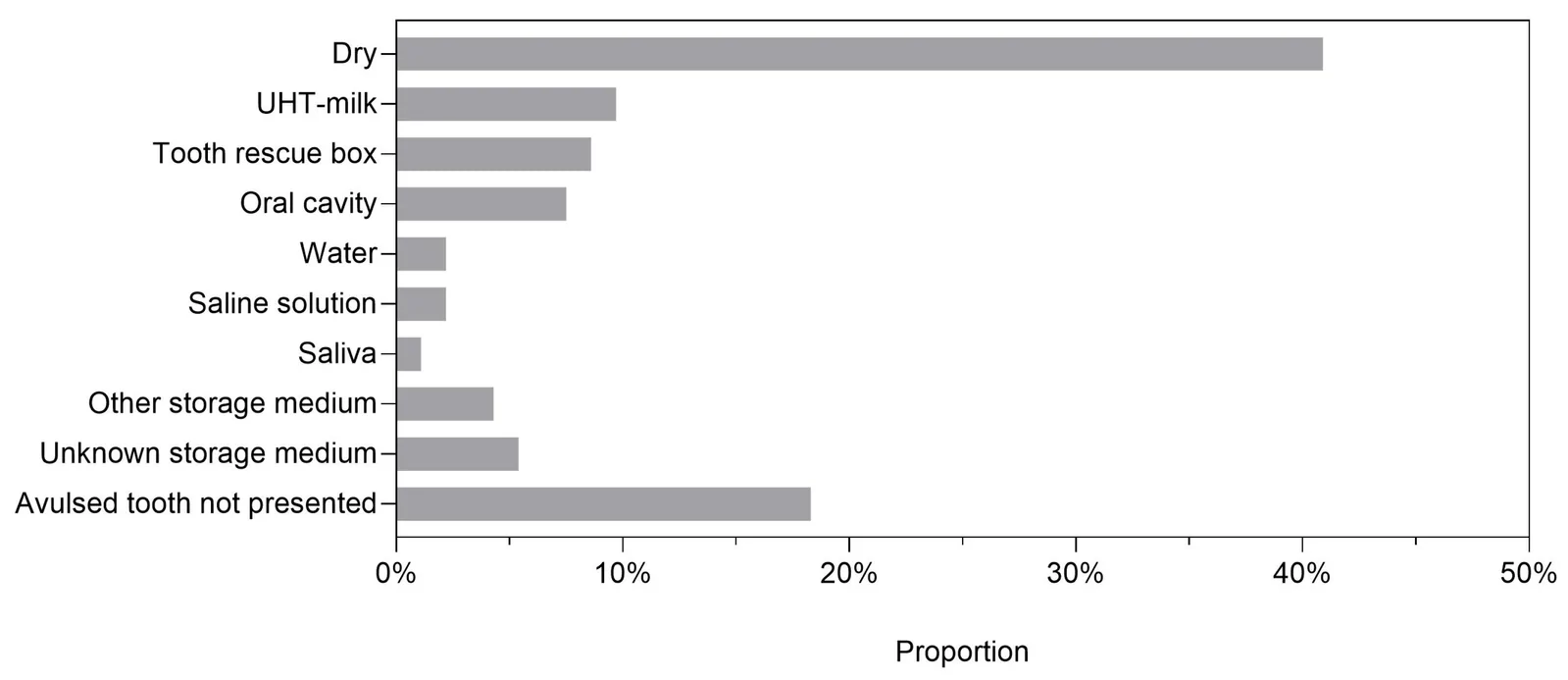
Distribution of initial storage conditions among cases involving avulsion of permanent teeth.

Among the 93 cases involving avulsion of at least one permanent tooth, dry storage was documented in 38 cases (40.9%) (Figure 4). Information on the extraoral dry time was missing in one of these cases. In the remaining 37 cases, the median extraoral dry time was 60 min (IQR: 25–90 min).

### 3.11. Treatment Measures

A total of 2530 therapeutic interventions were recorded for 2432 traumatized teeth. The most common initial treatment procedures were splinting (36.5%) and restoration or coverage (27.0%), followed by repositioning (11.3%). Less frequently performed interventions included fragment reattachment (5.1%), direct pulp capping (4.6%), replantation (3.9%), and placement of an intracanal medicament (3.0%). The least common procedures were indirect pulp capping (2.8%), tooth extraction (2.8%) and removal of tooth fragments (1.6%).

When stratified by dentition, splinting (45.9%) and restorations (33.9%) were the most common procedures in permanent teeth, whereas repositioning (10.2%) and extractions (9.1%) were most frequently performed in primary teeth. As multiple procedures could be applied to the same tooth, the cumulative percentages exceed 100% (Table 2).

## 4. Discussion

Understanding the injury characteristics and clinical presentation of TDIs is essential for optimizing their diagnosis, documentation and clinical management. The RDTR was initiated in 2016 to systematically record dental trauma cases presenting to the Center of Dental Traumatology at University Hospital Regensburg, which provides continuous 24/7 interdisciplinary dental trauma care in Eastern Bavaria. Although a national registry covering a wide range of traumatic medical injuries has existed in Germany for more than 30 years [19], comparable structured databases specifically dedicated to dentoalveolar traumatology have not yet been established. The RDTR addresses this gap by providing a methodological framework for standardized data collection, centralized data management and quality-controlled documentation in routine emergency care [15]. Based on the first 1000 eligible cases recorded during the initial study period, the present study describes the demographics, injury characteristics, diagnostic findings and initial management of TDIs. The broad age range and substantial number of cases enabled detailed descriptive analyses and comparisons across clinically relevant patient and injury characteristics.

The findings should be interpreted in consideration of the study design and clinical setting. As a single-center study conducted at a tertiary-care dental trauma center, the cohort reflects the distribution of TDI cases presenting to this specialized setting rather than the broader population. Accordingly, the findings cannot be used to estimate the incidence or risk of TDIs in Eastern Bavaria, and age- and sex-related patterns should be understood as characteristics of the registry cohort rather than population-level differences.

The composition of the cohort may also have been influenced by referral and ascertainment patterns. More complex injuries may have been preferentially referred to the center, whereas less severe injuries may have been managed elsewhere or may not have resulted in professional care-seeking. Conversely, the structured and detailed documentation used in the registry may have facilitated the identification of minor injuries. Healthcare-seeking behavior, oral health literacy, insurance status, and migration background may have further influenced which patients presented to the center and were therefore included in the registry [20].

The characteristics of the center itself may also have influenced management and treatment options. The absence of a specialized pediatric dentistry unit and the limited availability of sedation or general anesthesia may have affected the management of young or uncooperative children and, in some cases, the therapeutic options available.

The predefined inclusion of the first 1000 eligible cases resulted in the exclusion of 10 additional eligible cases registered in December 2020. Although this represents only a small proportion of the eligible cohort, a minor temporal selection effect on the overall descriptive findings cannot be excluded. Seasonal analyses were therefore restricted to the three fully represented calendar years (2017–2019). Year-specific distributions did not indicate a consistent secular pattern; nevertheless, potential changes in case ascertainment during the early implementation phase of the registry should be considered when interpreting the pooled seasonal distribution.

Finally, 21 cases represented recurrent TDIs in patients who had already contributed a case to the dataset. Because each traumatic event was analyzed as a separate case, observations were not entirely independent, and individual patient characteristics may therefore be represented more than once in case-level distributions.

The age distribution of presenting cases showed the highest frequencies among children aged 2–4 and 7–9 years, consistent with previous reports [21,22,23,24]. The first peak may be related to limited motor coordination during early walking, whereas the second may reflect increasing independence and greater exposure to school and playground activities [25,26,27]. Although previous studies have reported that 71–92% of TDIs occur before 19 years of age [6], the corresponding proportion was lower in the present cohort (60.9%). This difference may reflect the broader age range represented in the registry, as well as age-related referral patterns and healthcare-seeking behavior in a tertiary-care setting. The observed age distribution therefore reflects the characteristics of cases presenting to the center rather than age-specific incidence or risk in the underlying population.

Sex is considered a predisposing factor for TDIs with male predominance consistently reported in the literature, with female-to-male case ratios ranging from 1:1.4 to 1:3.8 [3,6,18,28,29,30,31]. In the present study, males accounted for 61.6% of the cohort, corresponding to a female-to-male case ratio of 1:1.6. This ratio reflects the distribution of cases within the cohort rather than population-based prevalence and is consistent with ratios reported in other German hospital-based studies [11,13,14]. Although males comprised the majority of registry cases overall, the middle-aged and older adult groups accounted for larger proportions of female than male registry cases. This pattern may partly reflect the greater frequency of falls among women aged 65 years and older [32]. Fall-related factors may contribute to the higher proportion of female cases among older adults, as falls are an important cause of injury in this population [33,34]. Risk factors for fall-related injuries may also be relevant among middle-aged women [35]. However, the occurrence and characteristics of TDIs in middle-aged and older adults have received comparatively little attention in the literature [36]. Further research in these age groups is therefore warranted, particularly in the context of demographic aging.

Temporal patterns of TDIs may be influenced by environmental and societal factors. Seasonal distributions have varied across studies, potentially reflecting differences in climate, outdoor activity, lifestyle, cultural practices, and school holidays. Nevertheless, some studies have found no significant association between season and TDI occurrence, suggesting that seasonal patterns may be context-dependent [4,29,37,38,39,40,41,42,43,44,45]. In the present cohort, injuries occurred most frequently on Saturdays, followed by Sundays and Fridays, consistent with a previous German study [13]. Although some authors have reported higher frequencies on weekdays [4,46], studies conducted at emergency care settings have observed a concentration of cases on Fridays and weekends [47,48,49], which may partly reflect differences in case ascertainment related to service availability. The largest proportion of injuries with a documented time occurred between 15:00 and 20:00, in line with previous studies [50,51,52]. By documenting injury and presentation times separately, the RDTR also enabled assessment of treatment timeliness: 84.1% of the cases sought care within the first 24 h after injury, with nearly half (49.5%) presenting within two hours of the traumatic event. These proportions were relatively high compared with previous reports [11,29,33,38,42,53,54,55,56]. Nevertheless, delayed presentation remains clinically relevant because many TDIs require time-sensitive management [24,57].

Another relevant parameter is the location at which the injury occurred. Nearly one-third of TDIs occurred at home, consistent with previous reports, particularly among young children [25,58,59,60,61]. Road traffic environments (9.8%) and sidewalks (6.0%) were the next most frequently documented locations. The lower proportion of traffic-related injuries compared with some cohorts from developing countries may reflect regional differences in urban infrastructure, traffic patterns, and road safety regulations [31,62].

Comparisons of TDI etiology across studies are complicated by the frequent combination of injury mechanism and activity at the time of trauma into a single category. By recording these variables separately, the RDTR provided a more differentiated description of trauma circumstances. Falls were the leading mechanism (67.8%), followed by blows (14.8%) and collisions (9.6%), consistent with previous reports [11,29,30,38,48,52]. However, the specific circumstances and contexts in which falls occurred remain insufficiently characterized in much of the literature [23,63,64].

Cycling was the most frequently documented activity at the time of injury (18.6%), followed by playing (17.1%) and sports (13.2%), consistent with the previous literature [29,30,65,66]. The increasing use of bicycles for transportation and recreation in Western Europe may contribute to changing TDI patterns [67,68]. From a preventive perspective, these findings emphasize the importance of protective measures during high-risk activities. The distribution of injury-related activities may also differ according to sex. In the present study, sports-related injuries and physical assaults accounted for a relatively larger proportion of male registry cases than female registry cases, a pattern also described in previous reports [29,30,38]. In contrast, walking or running and epilepsy- or syncope-related injuries represented a relatively larger proportion of female registry cases than male registry cases.

TDIs are frequently described as involving a single tooth [18,69], although the number of affected teeth per patient varies considerably across studies. In this study, the mean number of affected teeth was 2.4 per case, exceeding the values reported in comparable studies, possibly because complex cases are referred to a specialized multidisciplinary trauma center [3,30,33,52,70]. Maxillary central incisors remained the most frequently affected teeth in both dentitions, consistent with their anatomical susceptibility [49,61,71,72,73]. However, their proportions were lower than previously reported [45,56,74,75], accounting for 58.7% of injured primary teeth and 54.9% of injured permanent teeth. This may reflect the systematic documentation of posterior as well as anterior teeth. The overall left–right distribution was largely symmetrical, in agreement with previous studies [14,24,45,76,77,78].

The distribution of injury types differed between dentitions. Luxation injuries were documented in 92.8% of traumatized primary teeth and 69.8% of permanent teeth, whereas hard-tissue injuries were documented in 14.2% and 57.0%, respectively. These diagnostic categories were not mutually exclusive because a tooth could sustain both periodontal and hard tissue injuries. The predominance of luxation injuries in primary teeth is commonly attributed to the greater elasticity of the alveolar bone and periodontal ligament in young children, which favors displacement rather than fracture [11,12,18,29,42,63,79,80,81].

Among hard tissue injuries, enamel–dentin fractures were the most common type in both dentitions, accounting for 64.5% of all fractured teeth in both dentitions, including uncomplicated (52.4%) and complicated crown fractures (12.1%), consistent with previous studies [3,13,33]. The registry also captured trauma-related damage to existing restorations and fillings, which is not specifically represented in current trauma classifications or ICD systems. Its inclusion therefore provides a more comprehensive description of hard-tissue damage, particularly in older patients.

Root fractures are relatively uncommon among TDIs, with reported frequencies ranging from 0.5–7% in permanent teeth and 2–4% in the primary teeth [82]. In this study, however, root fractures accounted for a proportionally higher share of fractures in the primary (17.3%) than in permanent teeth (5.8%), consistent with previous observations [24,83]. These percentages refer to the distribution within fractured teeth and should not be directly compared with studies reporting root fractures as a proportion of all traumatized teeth. Physiological root resorption has been proposed as a potential contributor to root fracture susceptibility in primary teeth [84]. Notably, nearly half of the primary-tooth root fractures in this cohort involved the coronal third, whereas previous studies predominantly reported middle- and apical-third fractures [85,86]. In permanent teeth, the middle third was most frequently affected, consistent with previous reports [87,88].

In the present cohort, subluxation was the most frequently documented luxation type in both primary (38.2%) and permanent teeth (41.1%). It was followed by lateral luxation in the primary teeth (26.9%) and concussion in the permanent teeth (26.7%). The exact luxation subtype could not be determined in a small proportion of cases (4.3% in primary and 2.5% in permanent teeth), potentially because clinical position of the affected tooth had changed before presentation. An important feature of this registry is the detailed documentation of the injury extent. Although some studies have reported injury severity, structured and quantitative recording of displacement parameters is rarely available [24,75].

For avulsed permanent teeth, extraoral dry time is an important prognostic factor, as prolonged dry storage increases the risk for pathological root resorption [17,89,90,91,92]. Although some form of preservation was attempted in a high proportion of cases involving avulsed permanent teeth, tooth rescue box was used in only 8.6% of cases, while dry storage was documented in 40.9%. Among cases with available information, the median extraoral dry time was 60 min (IQR: 25–90 min). These findings indicate that, despite attempts at pre-hospital preservation, avulsed teeth were frequently exposed to potentially unfavorable storage conditions. Similarly, another study reported that only 20% of replanted teeth had been stored appropriately [93], while comparable delays in replantation have been described in previous studies [47,79,94].

Management of TDIs depends on dentition, injury type and patient-related factors such as age and cooperation, particularly in children [17,95]. In primary dentition, recommendations are largely consensus-based because of limited available evidence [95]. Many luxation injuries can be managed conservatively, while extraction, repositioning, or splinting may be required [24,44,47,85,86,95,96,97,98,99,100]. In the present cohort, repositioning and extraction were the most frequently recorded initial interventions; however, only 138 interventions were documented across 528 affected primary teeth. Apart from one case, no vital pulp therapy was performed in primary teeth, although such interventions have been occasionally reported in the literature [80,98,101]. This relatively low number may reflect the young age and associated cooperation challenges, as well as the absence of specialized pediatric dentistry unit.

In contrast, 2392 interventions were recorded across 1904 affected permanent teeth, with more than one intervention possible per tooth. Treatment was predominantly characterized by splinting and restorative procedures, reflecting luxation and hard tissue injuries and the resulting need for stabilization and restoration respectively [17,102]. Splinting has also been reported as the most frequent initial intervention in a comparable hospital-based emergency setting [33], although lower proportions have been observed in other studies [28,103]. Such variation likely reflects differences in injury severity, referral patterns, institutional structure, and local treatment protocols. Overall, the observed differences in treatment patterns between primary and permanent teeth may reflect differences in their respective injury profiles and clinical management considerations.

Despite the known importance of documenting TDI cases [61,104], documentation failures remain a challenge in emergency settings. In the present study, missing data were observed in both patient-reported variables (e.g., time of injury, extra-alveolar dry time, storage conditions) and clinical findings such as sensibility testing, particularly in younger patients and in the primary dentition. From a patient perspective, incomplete reporting is likely related to acute stress, pain, and limited recall of trauma-related details. From a clinician perspective, documentation gaps may arise from the time constraints in emergency settings as well as limited patient cooperation, especially in young or anxious children. Previous studies have also suggested that insufficient training and documentation systems not optimized for rapid, standardized data entry may contribute to missing or inconsistent data. In this context, continuous education and the implementation of simplified, digital, and clinician-friendly registry systems that enable rapid and structured data entry may help improve documentation quality in acute settings [105,106].

## 5. Conclusions

The RDTR demonstrates the feasibility and value of structured, quality-controlled data collection for the systematic characterization of TDIs in a tertiary hospital-based emergency care setting. The registry captures clinically relevant information on presenting cases, injury patterns, treatment strategies, and injury-specific parameters, providing a comprehensive basis for identifying areas for further research and potential improvement in trauma care. In particular, the documented pre-hospital storage conditions of avulsed permanent teeth and identified documentation gaps highlight opportunities for targeted improvement in clinical practice and data collection. Continued registry-based data collection and expansion to multicenter settings may strengthen the evidence base for the management of traumatic dental injuries and facilitate comparative research.

## Figures and Tables

**Table 1:** Distribution of fracture and luxation types in the permanent and primary teeth.

**Type of Fracture**	**Permanent**			**Primary**			**All Teeth**		
	**n**	**% Within Permanent**	**% of All** **Affected Teeth**	**n**	**% Within Primary**	**% of All** **Affected Teeth**	**n**	**% Within Fractures**	**% of All Injuries**
Infraction	81	7.5	4.3	6	8.0	1.1	87	7.5	3.6
Enamel fracture	162	14.9	8.5	14	18.7	2.7	176	15.2	7.2
Enamel & dentin fracture	712	65.6	37.4	36	48.0	6.8	748	64.5	30.8
- with pulp involvement (complicated crown fracture)	130	12.0	6.8	10	13.3	1.9	140	12.1	5.8
- without pulp involvement (uncomplicated crown fracture)	582	53.6	30.6	26	34.7	4.9	608	52.4	25.0
Crown-root fracture	55	5.1	2.9	4	5.3	0.8	59	5.1	2.4
- with pulp involvement (complicated crown-root fracture)	35	3.2	1.8	3	4.0	0.6	38	3.3	1.6
- without pulp involvement (uncomplicated crown-root fracture)	18	1.7	0.9	0	0	0	18	1.6	0.7
- not documented	2	0.2	0.1	1	1.3	0.2	3	0.3	0.1
Root fracture	63	5.8	3.3	13	17.3	2.5	76	6.6	3.1
-coronal third	9	0.8	0.5	6	8.0	1.1	15	1.3	0.6
-middle third	32	2.9	1.7	3	4.0	0.6	35	3.0	1.4
-apical third	22	2.0	1.2	4	5.3	0.8	26	2.2	1.1
Vertical root fracture	13	1.2	0.7	2	2.7	0.4	15	1.3	0.6
Damage to prosthetic restoration	45	4.1	2.4	1	1.3	0.2	46	4.0	1.9
Damage to filling	14	1.3	0.6	0	0	0	14	1.2	0.6
Total fractured teeth	1085	100	57.0	75	100	14.2	1160	100	47.7
(All affected teeth)	(1904)		(100)	(528)		(100)	(2432)		(100)
**Type of Luxations**	**Permanent**			**Primary**			**All Teeth**		
	**n**	**% Within Permanent**	**% of All** **Affected Teeth**	**n**	**% Within Primary**	**% of All** **Affected Teeth**	**n**	**% Within Luxations**	**% of All Injuries**
Concussion	355	26.7	18.6	34	6.9	6.4	389	21.4	16.0
Subluxation	546	41.1	28.7	187	38.2	35.4	733	40.3	30.1
Lateral luxation	189	14.2	9.9	132	26.9	25.0	321	17.6	13.2
Extrusion	40	3.0	2.1	18	3.7	3.4	58	3.2	2.4
Intrusion	26	2.0	1.4	35	7.1	6.6	61	3.3	2.5
Avulsion	139	10.5	7.3	63	12.9	11.9	202	11.1	8.3
No information	34	2.5	1.8	21	4.3	4.0	55	3.1	2.3
Total luxated teeth	1329	100	69.8	490	100	92.8	1819	100.0	74.8
(All affected teeth)	(1904)		(100)	(528)		(100)	(2432)		(100)

**Table 2:** Distribution of initial therapeutic measures for traumatic dental injuries. “No information” was not included in the total number of therapeutic interventions.

Therapeutic Intervention	Permanent		Primary		All Teeth	
	n	% of All Affected Teeth	n	% of All Affected Teeth	n	% of All Affected Teeth
Splinting	873	45.9	15	2.8	888	36.5
Restoration or coverage	645	33.9	11	2.1	656	27.0
Repositioning	222	11.7	54	10.2	276	11.3
Fragment reattachment	123	6.5	-	-	123	5.1
Direct pulp capping	110	5.8	1	0.2	111	4.6
Replantation	94	4.9	-	-	94	3.9
Intracanal medicament	73	3.8	-	-	73	3.0
Indirect pulp capping	69	3.6	-	-	69	2.8
Pulpectomy	65	3.4	-	-	65	2.7
Removal of tooth fragment(s)	32	1.7	7	1.3	39	1.6
Tooth extraction	21	1.1	48	9.1	69	2.8
Partial pulpotomy	27	1.4	-	-	27	1.1
Smoothing sharp edges	27	1.4	2	0.4	29	1.2
Full pulpotomy	11	0.6	-	-	11	0.5
No information	2	0.1	1	0.2	3	0.1

## Data Availability

The data supporting the findings of this study are available from the corresponding author upon reasonable request.

## Supplementary Materials

The following supporting information can be downloaded at https://www.mdpi.com/article/10.3390/jcm15207947/s1: Supplementary Figure S1: Temporal distribution of traumatic dental injuries (TDIs): (**A**) distribution by day of the week and (**B**) distribution by time of day; Supplementary Figure S2: Time from traumatic dental injury to clinical presentation among cases presenting within three days, with an hourly breakdown; Supplementary Figure S3: Distribution of traumatized teeth by tooth number, presented separately for (**A**) permanent teeth and (**B**) primary teeth; Supplementary Figure S4: Distribution of fracture and luxation injuries in primary and permanent teeth; Supplementary Figure S5: Distribution of mobility grades among teeth with subluxation injuries; Supplementary Figure S6: Extent of displacement by luxation injury type. Median displacement values (mm) are presented for intrusion, extrusion, and lateral luxation injuries in permanent and primary teeth. Bars represent median values, and error bars indicate the interquartile range (IQR; 25th–75th percentiles); Supplementary Table S1: REporting of studies Conducted using Observational Routinely collected health Data (RECORD) checklist [15]; Supplementary Table S2: Plausibility checks applied during data quality assurance, their outcomes, and excluded cases; Supplementary Table S3: Seasonal distribution of TDI occurrence during the complete calendar years 2017–2019; Supplementary Table S4: Available and missing information for key case-level and tooth-level variables; Supplementary Table S5: Case-level distribution of activity at the time of trauma, injury mechanism, and injury location by sex, including descriptive proportion ratios; Supplementary Table S6: Baseline pulp sensibility and percussion findings in traumatized permanent and primary teeth; Supplementary Table S7: Case-level distribution of radiographic imaging modalities by age group.

## Abbreviations

The following abbreviations are used in this manuscript:

CBCT	Cone-beam computed tomography
IQR	Interquartile range
OPG	Orthopantomogram
RDTR	Regensburg Dental Trauma Registry
RECORD	REporting of studies Conducted using Observational Routinely collected health Data
SD	Standard deviation
TDI	Traumatic dental injury
