# Supplementary material for: Traumatic Dental Injuries at a Tertiary Dental Trauma Center in Eastern Bavaria: Findings from the Regensburg Dental Trauma Registry
Source: J Clin Med. 2026 Oct 14;15(20):7947. doi: 10.3390/jcm15207947 (PMC13706110; doi:10.3390/jcm15207947)
Supplement: Supplementary file 1 [file jcm-15-07947-s001.zip › jcm-4500382-supplementary.pdf]

Supplementary Materials

Supplementary Figures

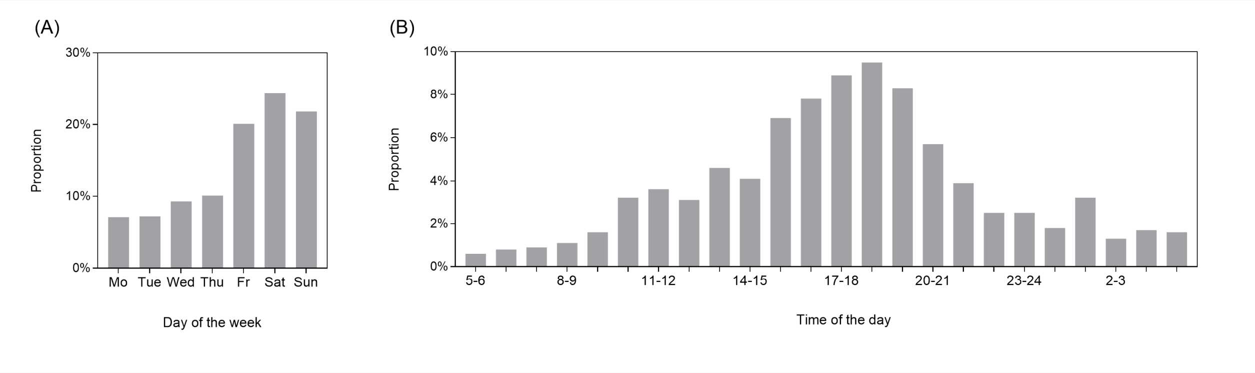

Supplementary Figure S1. Temporal distribution of traumatic dental injuries (TDIs): (A) distribution by day of the week and (B) distribution by time of day.

Supplementary Figures

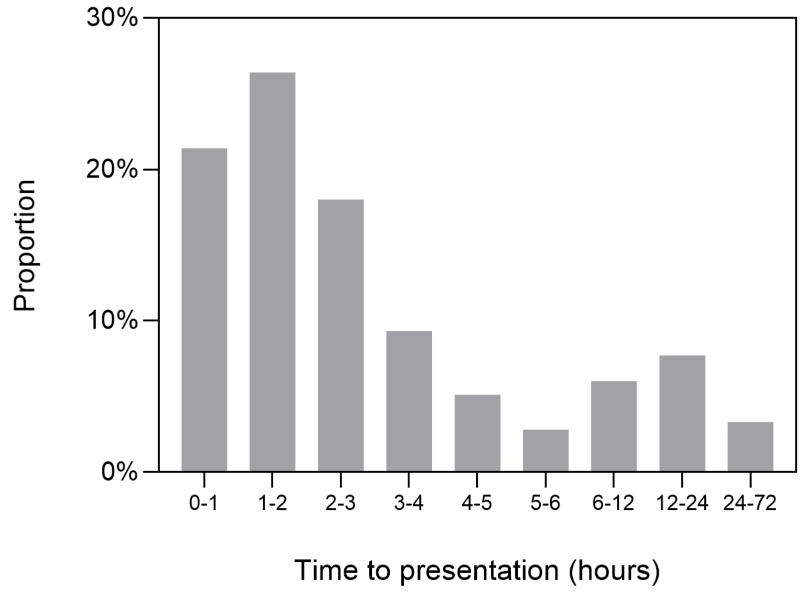

Supplementary Figure S2. Time from traumatic dental injury to clinical presentation among cases presenting within three days, with an hourly breakdown.

Supplementary Figures

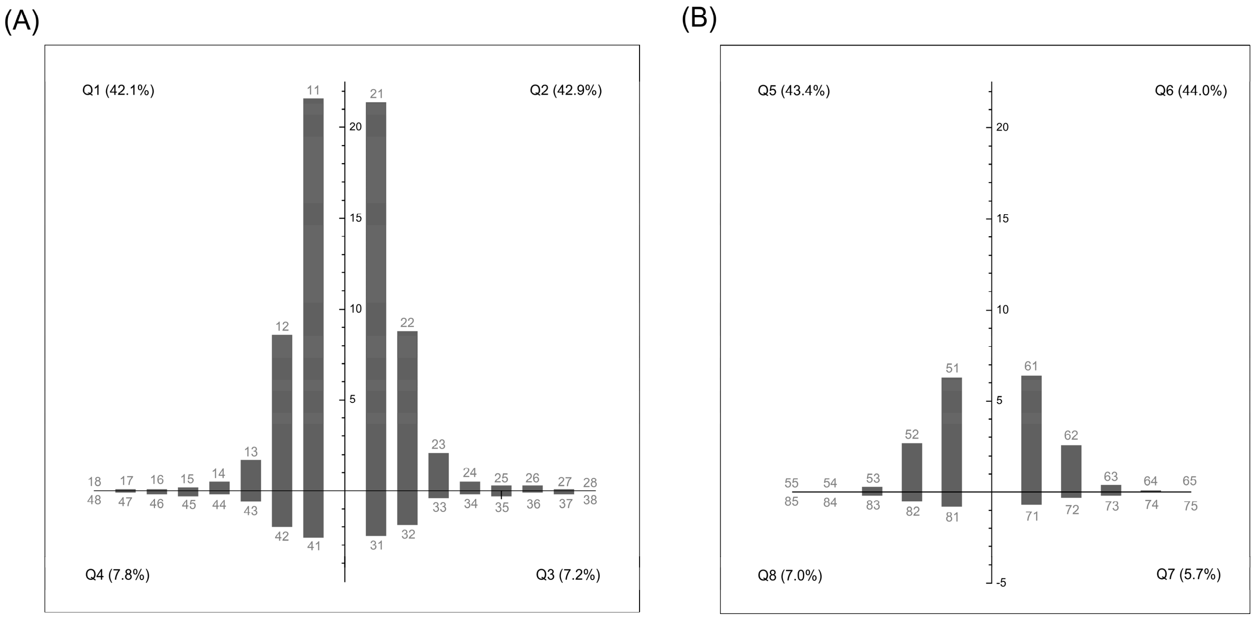

Supplementary Figure S3. Distribution of traumatized teeth by tooth number, presented separately for (A) permanent teeth and (B) primary teeth.

Supplementary Figures

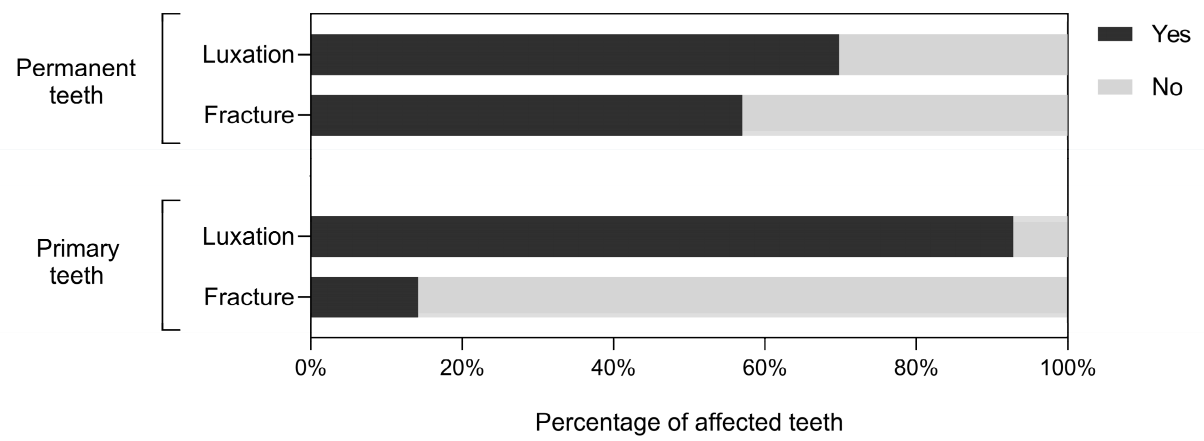

Supplementary Figure S4. Distribution of fracture and luxation injuries in primary and permanent teeth.

Supplementary Figures

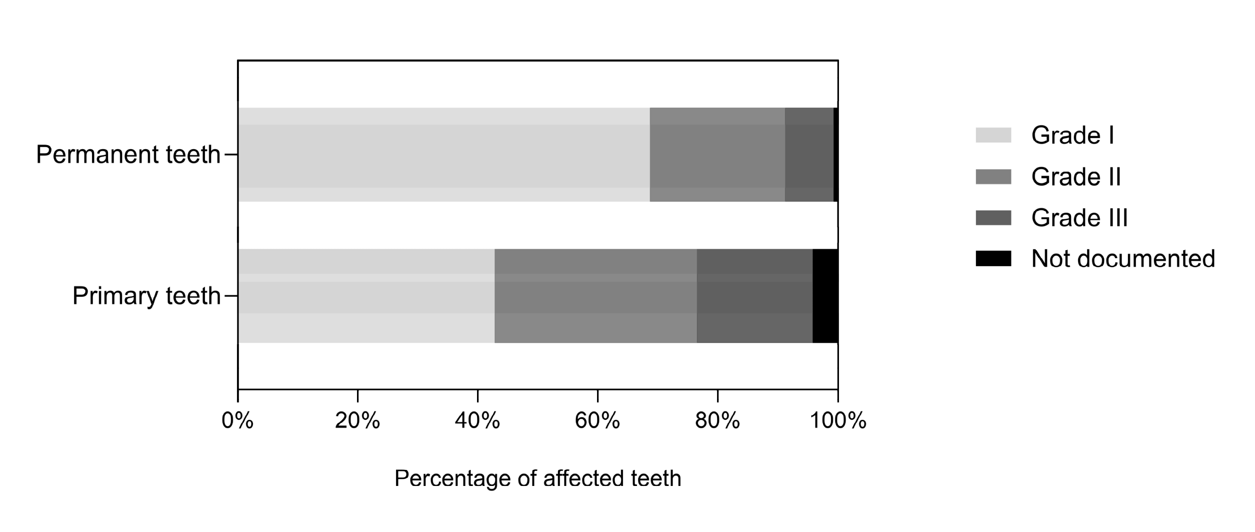

Supplementary Figure S5. Distribution of mobility grades among teeth with subluxation injuries.

Supplementary Figures

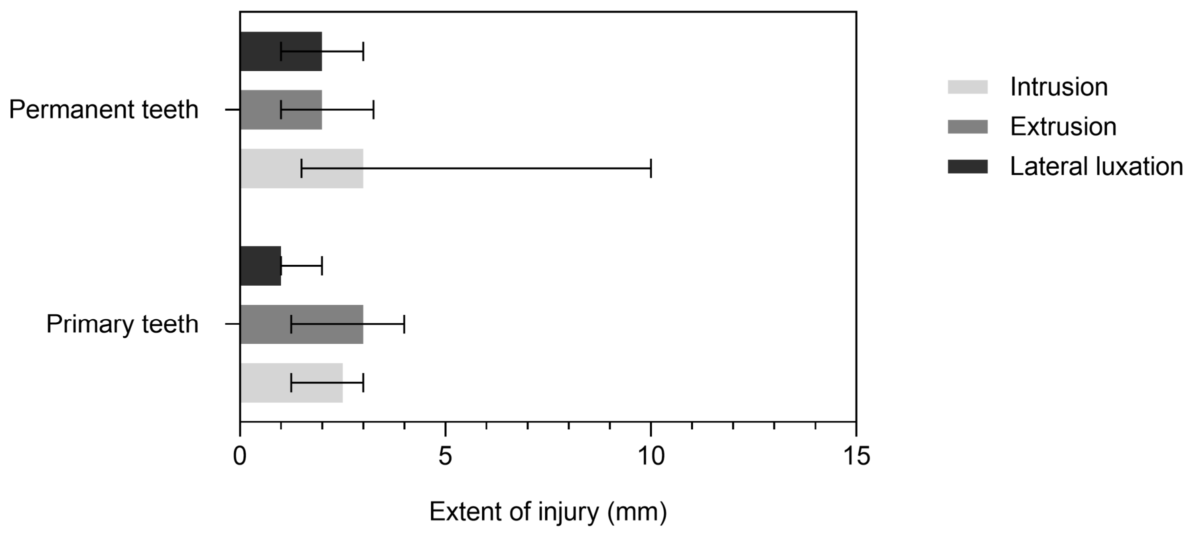

Supplementary Figure S6. Extent of displacement by luxation injury type. Median displacement values (mm) are presented for intrusion, extrusion, and lateral luxation injuries in permanent and primary teeth. Bars represent median values, and error bars indicate the interquartile range (IQR; 25th–75th percentiles).

Supplementary Tables

Supplementary Table S1. REporting of studies Conducted using Observational Routinely collected health Data (RECORD) checklist

Section / Item	Item No.	Recommendation	Page & Line #
**Title and abstract**	1	(a) Indicate the study's design with a commonly used term in the title or the abstract	p1, #20
		(b) Provide in the abstract an informative and balanced summary of what was done and what was found	p1, #20-39
		(c) The type of data used should be specified in the title or abstract. When possible, the name of the databases used should be included. If applicable, the geographic region and time frame within which the study took place should be reported in the title or abstract. If linkage between databases was conducted for the study, this should be clearly stated in the title or abstract.	p1, #1-4, #20-25
**Introduction**			
Background/rationale	2	Explain the scientific background and rationale for the investigation being reported	p2, #61-86
Objectives	3	State specific objectives, including any prespecified hypotheses	p2, #87-94
**Methods**			
Study design	4	Present key elements of study design early in the paper	p3, #97-114
Setting	5	Describe the setting, locations, and relevant dates, including periods of recruitment, exposure, follow-up, and data collection	p4, Figure 1
Participants	6	RECORD 6.1: The methods of study population selection (such as codes or algorithms used to identify subjects) should be listed in detail. If this is not possible, an explanation should be provided.	p4, #101-108
		RECORD 6.2: Any validation studies of the codes or algorithms used to select the population should be referenced. If validation was conducted for this study and not published elsewhere, detailed methods and results of how study participants were identified from medical records should be provided.	p3, #107-108
		RECORD 6.3: If the study involved linkage of databases, consider use of a flow diagram or other graphical display to demonstrate the data linkage process, including the number of individuals with linked data at each stage.	NA
Variables	7	A complete list of codes and algorithms used to classify exposures, outcomes, confounders, and effect modifiers should be provided. If these cannot be reported, an explanation should be provided.	Detailed explanation is provided in another publication [15]
Data sources/measurement	8*	For each variable of interest, give sources of data and details of methods of assessment (measurement). Describe comparability of assessment methods if there is more than one group.	p3, #116-136
Bias	9	Describe any efforts to address potential sources of bias	p3, #116-118
Study size	10	Explain how the study size was arrived at	p3, #132-134
Quantitative variables	11	Explain how quantitative variables were handled in the	p4-p5, #150-175

		analyses. If applicable, describe which groupings were chosen and why	
Statistical methods	12	(a) Describe all statistical methods, including those used to control for confounding	p5, #177-181
		(b) Describe any methods used to examine subgroups and interactions	NA
		(c) Explain how missing data were addressed	Figure 1
		(d) Case-control study—If applicable, explain how matching of cases and controls was addressed. Cross-sectional study—If applicable, describe analytical methods taking account of sampling strategy.	NA
		(e) Describe any sensitivity analyses	NA
Data access and cleaning methods	12.1	Authors should describe the extent to which the investigators had access to the database population used to create the study population.	p3, #100-103
	12.2	Authors should provide information on the data cleaning methods used in the study.	p3, #119-127
Linkage	12.3	State whether the study included person-level, institutional-level, or other data linkage across two or more databases. The methods of linkage and methods of linkage quality evaluation should be provided.	NA
**Results**			
Participants	13*	Describe in detail the selection of the persons included in the study (i.e., study population selection), including filtering based on data quality, data availability, and linkage. The selection of included persons can be described in the text and/or by means of the study flow diagram.	p4, Figure 1
Descriptive data	14*	(a) Give characteristics of study participants (eg demographic, clinical, social) and information on exposures and potential confounders	p5, #186-191
		(b) Indicate number of participants with missing data for each variable of interest	Supplementary Table S4
		(c) Cohort study—Summarise follow-up time (eg, average and total amount)	NA
Outcome data	15*	Cohort study—Report numbers of outcome events or summary measures over time	NA
		Case-control study—Report numbers in each exposure category, or summary measures of exposure	NA
		Cross-sectional study—Report numbers of outcome events or summary measures	pp.5-12
Main results	16	(a) Give unadjusted estimates and, if applicable, confounder-adjusted estimates and their precision (eg, 95% confidence interval). Make clear which confounders were adjusted for and why they were included	NA
		(b) Report category boundaries when continuous variables were categorized	p4, #150-157
		(c) If relevant, consider translating estimates of relative risk into absolute risk for a meaningful time period	NA
Other analyses	17	Report other analyses done—eg analyses of subgroups and interactions, and sensitivity analyses	NA
**Discussion**			
Key results	18	Summarise key results with reference to study objectives	pp.12-16
Limitations	19	Discuss the implications of using data that were not created	p16, #613-623

		or collected to answer the specific research question(s). Include discussion of misclassification bias, unmeasured confounding, missing data, and changing eligibility over time.	
Interpretation	20	Give a cautious overall interpretation of results considering objectives, limitations, multiplicity of analyses, results from similar studies, and other relevant evidence	pp.12-16
Generalizability	21	Discuss the generalisability (external validity) of the study results	p12, #434-440
**Other information**			
Funding	22	Give the source of funding and the role of the funders for the present study and, if applicable, for the original study on which the present article is based	NA
Accessibility of protocol, raw data, and programming code	23	Authors should provide information on how to access any supplemental information such as the study protocol, raw data, or programming code.	p16-17, #637-659

Supplementary Table S2. Plausibility checks applied during data quality assurance, their outcomes, and excluded cases.

No.	Plausibility questions	Number of cases evaluated	Number of cases that were excluded	Reason for exclusion
1	The accident date must not be later than the date of the initial presentation.	-	-	-
2	The recorded accident time must not be later than the time of first presentation if both events occurred on the same day.	1	-	-
3	If the accident location is recorded as work, school, or kindergarten, the accident must be classified as an occupational, school, or commuting accident, respectively.	1	-	-
4	If fragment reattachment or tooth replantation was performed, the question regarding patient measures for preserving the tooth fragment should not be answered "No."<br>An exception may apply to crown-root fractures, in which the fragment may have been reattached without prior storage in a preservation medium.	10	-	-
5	If a soft tissue injury is documented, the type of soft tissue injury must also be specified.	2	-	-
6	At least one response option for radiographic examination must be selected. If no radiographic examination was performed, "None" should be recorded.	2	-	-
7	If splinting is documented as a treatment measure, the corresponding field indicating the number of splinted teeth must also be completed.	4	2	*Reason for exclusion:*<br>* 8 teeth are splinted in maxilla, only one mandibular tooth was registered.<br>* Alveolar fracture, splinting of primary teeth but no tooth related finding- which was considered implausible.
8	If the question regarding loss of consciousness is answered with 'Yes', then alcohol and/or drugs must also be marked	-	-	-
9	If "foetor alcoholicus" is recorded, alcohol must also be selected under "reason for impaired consciousness." The reverse is not necessarily required.	-	-	-
10	No permanent teeth for kids under 4 years old.	3	-	-
11	The number of splinted teeth must be at least	-	-	-

	equal to the number of traumatized teeth for which splinting was indicated as a therapeutic measure.			
12	The reported number of affected teeth must be specified and correspond to the number of teeth selected.	-	-	-
13	Avulsed teeth cannot have a positive vitality or percussion test result; only “not possible” is permitted for Questions 24 and 25.	6	-	-
14	Concussions always present with a positive percussion response.	-	-	-
15	Avulsed teeth are only replanted, not repositioned.	-	-	-
16	No pulpectomy should be performed without an intracanal dressing and a restoration/coverage.	-	-	-
17	No replantation or temporary storage in ‘Dentosafe’ should be performed for primary teeth.	-	-	-
18	No alveolar process fracture should be recorded for an isolated crown fracture or infraction.	-	-	-
19	For the question “Was the traumatized area treated with a splint?”, “No” cannot be selected if any traumatized tooth was splinted.	-	-	-
20	Teeth with an isolated enamel fracture should not undergo pulpotomy, pulpectomy, or replantation.	-	-	-

Supplementary Table S3. Seasonal distribution of TDI occurrence during the complete calendar years 2017–2019

Seasons	2017, *n* (%)	2018, *n* (%)	2019, *n* (%)	Total, *n* (%)
Winter (December - February)	50 (22.0)	50 (20.8)	50 (18.2)	150 (20.2)
Spring (March - May)	57 (25.1)	65 (27.1)	75 (27.4)	197 (26.6)
Summer (June - August)	75 (33.0)	54 (22.5)	81 (29.6)	210 (28.3)
Autumn (September - November)	45 (19.8)	71 (29.6)	68 (24.8)	184 (24.8)
Total	227 (100.0)	240 (100.0)	274 (100.0)	741 (100.0)

Supplementary Table S4. Available and missing information for key case-level and tooth-level variables

**Data are presented as n (%). Percentages were calculated using the total number of observations eligible for the respective variable as the denominator. Missingness includes observations recorded as "no information," "not documented," or otherwise unavailable.*

Variable	Denominator	n	Available, n (%)	Missing, n (%)
Case-level variables				
Time of injury	All cases	1000	892 (89.2)	108 (10.8)
Time to clinical presentation	All cases	1000	870 (87.0)	130 (13.0)
Activity at the time of injury	All cases	1000	830 (83.0)	170 (17.0)
Mechanism of injury	All cases	1000	933 (93.3)	67 (6.7)
Location of injury	All cases	1000	753 (75.3)	247 (24.7)
Alveolar process fracture status	All cases	1000	991 (99.1)	9 (0.9)
Extraoral dry time	Permanent-tooth avulsion cases involving dry storage	38	37 (97.4)	1 (2.6)
Tooth-level variables				
Cold sensibility	Permanent teeth	1904	1433 (75.3)	471 (24.7)
	Primary teeth	528	159 (30.1)	369 (69.9)
Percussion	Permanent teeth	1904	1454 (76.4)	450 (23.6)
	Primary teeth	528	244 (46.2)	284 (53.8)
Specific luxation injury type	Permanent luxated teeth	1329	1295 (97.4)	34 (2.6)
	Primary luxated teeth	490	469 (95.7)	21 (4.3)
Pulp involvement status in crown-root fractures	All teeth with crown-root fractures	59	56 (94.9)	3 (5.1)
Initial treatment information	All affected teeth	2432	2429 (99.9)	3 (0.1)

		Overall, n (%)	Male, n (%)	Female, n (%)	Descriptive fold ratio
**Activity at the time of trauma**	Sports	132 (13.2)	96 (15.6)	36 (9.4)	1.7 M/F
	Playing	171 (17.1)	100 (16.2)	71 (18.5)	—
	Cycling	186 (18.6)	120 (19.5)	66 (17.2)	—
	Walking or running	110 (11.0)	50 (8.1)	60 (15.6)	1.9 F/M
	Physical assault	57 (5.7)	54 (8.8)	3 (0.8)	11.2 M/F
	Leisure activity or partying	57 (5.7)	29 (4.7)	28 (7.3)	—
	Syncope, stroke, or epilepsy	34 (3.4)	14 (2.3)	20 (5.2)	2.3 F/M
	Work-related activity	27 (2.7)	20 (3.2)	7 (1.8)	1.8 M/F
	Motorcycling	12 (1.2)	7 (1.1)	5 (1.3)	—
	Showering or bathing	11 (1.1)	6 (1.0)	5 (1.3)	—
	Eating or drinking	8 (0.8)	5 (0.8)	3 (0.8)	—
	Driving	6 (0.6)	4 (0.6)	2 (0.5)	—
	Other	19 (1.9)	9 (1.5)	10 (2.6)	—
	Not documented	170 (17.0)	102 (16.6)	68 (17.7)	—
**Mechanism of Injury**	Fall	678 (67.8)	370 (60.1)	308 (80.2)	1.3 F/M
	Blow	148 (14.8)	119 (19.3)	29 (7.6)	2.6 M/F
	Collision	96 (9.6)	78 (12.7)	18 (4.7)	2.7 M/F
	Other, including food-related incidents	11 (1.1)	7 (1.1)	4 (1.0)	—
	Unknown or not documented	67 (6.7)	42 (6.8)	25 (6.5)	—
**Location of Injury**	Playground	25 (2.5)	16 (2.6)	9 (2.3)	—
	Sports field	44 (4.4)	36 (5.8)	8 (2.1)	2.8 M/F
	Sports hall	34 (3.4)	25 (4.1)	9 (2.3)	—
	Road or street	98 (9.8)	66 (10.7)	32 (8.3)	—
	Sidewalk or pedestrian pathway	60 (6.0)	29 (4.7)	31 (8.1)	1.7 F/M
	Domestic environment	330 (33.0)	186 (30.2)	144 (37.5)	1.2 F/M
	Bar, restaurant, or discotheque	23 (2.3)	20 (3.2)	3 (0.8)	4.2 M/F
	Park, natural environment, or mountain	16 (1.6)	10 (1.6)	6 (1.6)	—
	School	14 (1.4)	10 (1.6)	4 (1.0)	—
	Kindergarten	8 (0.8)	5 (0.8)	3 (0.8)	—
	Workplace	22 (2.2)	17 (2.8)	5 (1.3)	2.1 M/F
	Swimming pool	30 (3.0)	20 (3.2)	10 (2.6)	—
	Public place	26 (2.6)	15 (2.4)	11 (2.9)	—
	Other	23 (2.3)	11 (1.8)	12 (3.1)	—

	Not documented	247 (24.7)	150 (24.4)	97 (25.3)	—
	**Total**	**1000 (100.0)**	**616 (100.0)**	**384 (100.0)**	

Supplementary Table S5. Case-level distribution of activity at the time of trauma, injury mechanism, and injury location by sex, including descriptive proportion ratios.

Supplementary Table S6. Baseline pulp sensibility and percussion findings in traumatized permanent and primary teeth

Test	Type of tooth	Available results, n (% of all teeth)	Positive, n (% of available results)	Negative, n (% of available results)	No information, n (% of all teeth)
Sensibility	Permanent (n = 1904)	1433 (75.3)	1220 (85.1)	213 (14.9)	471 (24.7)
	Primary (n = 528)	159 (30.1)	123 (77.4)	36 (22.6)	369 (69.9)
	All teeth (n = 2432)	1592 (65.5)	1343 (84.4)	249 (15.6)	840 (34.5)
Percussion	Permanent (n = 1904)	1454 (76.4)	816 (56.1)	638 (43.9)	450 (23.6)
	Primary (n = 528)	244 (46.2)	140 (57.4)	104 (42.6)	284 (53.8)
	All teeth (n = 2432)	1698 (69.8)	956 (56.3)	742 (43.7)	734 (30.2)

Supplementary Table S7: Case-level distribution of radiographic imaging modalities by age group.

Age group	Total, n	No radiographic imaging, n (%)	Periapical radiograph, n (%)	OPG, n (%)	Other in-house imaging, n (%)	CBCT, n (%)
Toddlers (0 - 3)	142	132 (93.0)	6 (4.2)	2 (1.4)	0 (0.0)	0 (0.0)
Children (4 - 12)	354	199 (56.2)	101 (28.5)	66 (18.6)	2 (0.6)	0 (0.0)
Adolescents (13 - 18)	113	37 (32.7)	46 (40.7)	39 (34.5)	3 (2.7)	0 (0.0)
Young adults (19 - 27)	159	47 (29.6)	50 (31.4)	63 (39.6)	5 (3.1)	1 (0.6)
Adults (28 - 65)	210	40 (19.0)	83 (39.5)	108 (51.4)	8 (3.8)	0 (0.0)
Older adults (≥ 66)	22	4 (18.2)	8 (36.4)	10 (45.5)	0 (0.0)	0 (0.0)
Total	1000	459 (45.9)	294 (29.4)	288 (28.8)	18 (1.8)	1 (0.1)
